# A Mini-Review of *Ixodes* Ticks Climate Sensitive Infection Dispersion Risk in the Nordic Region

**DOI:** 10.3390/ijerph17155387

**Published:** 2020-07-27

**Authors:** Bob E. H. van Oort, Grete K. Hovelsrud, Camilla Risvoll, Christian W. Mohr, Solveig Jore

**Affiliations:** 1 CICERO Center for International Climate Research, P.O. Box 1129, Blindern, 0318 Oslo, Norway; 2 Nord University and Nordland Research Institute, P.O. Box 1490, 8049 Bodø, Norway; grete.hovelsrud@nord.no; 3 Nordland Research Institute, P.O. Box 1490, 8049 Bodø, Norway; cri@nforsk.no; 4 The Norwegian Institute of Bioeconomy Research, P.O. Box 115, 1431 Ås, Norway; christian.mohr@nibio.no; 5 Norwegian Public Health Institute, P.O. Box 222 Skøyen, 0213 Oslo, Norway; solveig.jore@fhi.no

**Keywords:** tick-borne diseases (TBD), climate-sensitive infections (CSIs), *Ixodes ricinus*, climate change, Nordic

## Abstract

Climate change in the Nordic countries is projected to lead to both wetter and warmer seasons. This, in combination with associated vegetation changes and increased animal migration, increases the potential incidence of tick-borne diseases (TBD) where already occurring, and emergence in new places. At the same time, vegetation and animal management influence tick habitat and transmission risks. In this paper, we review the literature on *Ixodes ricinus*, the primary vector for TBD. Current and projected distribution changes and associated disease transmission risks are related to climate constraints and climate change, and this risk is discussed in the specific context of reindeer management. Our results indicate that climatic limitations for vectors and hosts, and environmental and societal/institutional conditions will have a significant role in determining the spreading of climate-sensitive infections (CSIs) under a changing climate. Management emerges as an important regulatory “tool” for tick and/or risk for disease transfer. In particular, shrub encroachment, and pasture and animal management, are important. The results underscore the need to take a seasonal view of TBD risks, such as (1) grazing and migratory (host) animal presence, (2) tick (vector) activity, (3) climate and vegetation, and (4) land and animal management, which all have seasonal cycles that may or may not coincide with different consequences of climate change on CSI migration. We conclude that risk management must be coordinated across the regions, and with other land-use management plans related to climate mitigation or food production to understand and address the changes in CSI risks.

## 1. Introduction

In the Nordic region, projected climate change can be summarized as increasingly warmer and wetter weather, and a higher frequency of extreme weather events, including droughts, floods and cold and heat spells [1]. Vegetation and animals in the Arctic adapt to the changing conditions by following the shifting ecosystem conditions to higher elevations or latitudes, as new niches open up and the climate becomes less suitable in the southern regions, and as new invading species compete with existing species. Such shifts and migrations are not limited to vegetation and animals, but also include pathogens that may follow their vectors or hosts, which means new co-existences of both pathogens and vector species in new places. Especially the warmer and wetter conditions may enable such pathogens to survive further north than before, and find new hosts in northern species, although it is not a given that pathogens will also cause disease. On the other hand, heavy precipitation, flooding, and increased numbers of hot, dry and cold spells may limit their migration. The introduction of emerging or re-emerging zoonoses may pose new risks to both animal and human health. In this paper, we discuss the climate sensitivity of such zoonoses under the term “climate-sensitive infections (CSIs)”. This is currently not an official group of diseases, but is here defined as zoonotic infections causing diseases where the spread is sensitive to changes in the climate. This can be a direct effect, e.g., through re-emergence from melting permafrost, or an indirect effect, e.g., through vector (e.g., mosquitos, flies or ticks), hosts (e.g., rodents, cervids, hare, geese) or vegetation changes [2].

Recent research on the presence and distribution of reindeer pathogens in northern Norway [2,3], historical literature on reindeer husbandry and diseases in northern Scandinavia [4,5,6] and information from reindeer herders and resource managers on animal diseases highlighted six potentially significant CSIs: Anaplasmosis, babesiosis, parapox virus, tularemia, necrobacillosis and anthrax. In this paper we focus on a subset of these CSIs, transmitted by the *Ixodes ricinus* tick [7], namely anaplasmosis, babesiosis and tularemia. The bacterium *Anaplasma phagocytophilum* has been identified in such *I. ricinus* tick species, but also in *Ixodes persulcatus* in eastern Europe and Finland, and in other ticks within the genera *Dermacentor* and *Rhiplicephalus* [8,9]. However, in most of Europe including Sweden and Norway, *I. ricinus* is the main vector of anaplasmosis and babesiosis [10,11,12,13]. Tularemia in the Nordic countries is primarily a waterborne disease, and is mostly explained by the presence of mosquitoes [14,15], but on the European continent it is also [16,17], or even exclusively [18], associated with *Ixodid* ticks. We will therefore consider tularemia as a potential tick-borne disease (TBD). Symptoms for anaplasmosis and babesiosis in humans typically include muscle aches, fever, chills, weakness, lethargy, jaundice, nausea and loss of appetite, whilst for tularemia there is a broad spectrum of symptoms. In animals, symptoms can range from asymptomatic to fever, reduced milk production, weight loss, urination of blood, anemia, and even death. In the context of climate change, these infections are presumably sensitive, at some or all stages of the life cycle of the pathogens, vectors and hosts, to climate variables such as increased temperatures, precipitation, changing freeze–thaw cycles and snow cover. We argue that these diseases, anaplasmosis, babesiosis and tularemia (ABT), their main vector (*I. ricinus*) and their hosts provide a good example of how climate can influence the migration of CSIs.

## 2. Climate-Sensitive Infections: The Tick

Several authors have identified factors that contribute to the (changing) incidence and geographical distribution of diseases, including biotic and abiotic variables such as climate, vegetation, the presence of host animals, human land-use and land-use practices [12,13,19,20,21,22,23,24]. These variables are logically connected, as climate influences vegetation, which additionally is influenced by human management and grazing animals. Both ticks and hosts have preferences for certain types of vegetation, and their distribution depends both on the presence and continuity of suitable vegetations. For ticks, distribution also depends on the availability, mobility and management practices of the host animals. These different factors tend to vary spatially and seasonally and affect different stages of the vectors (in this case ticks) and hosts differently [11,25,26]. Finally, distribution depends on the responses of animal healthcare and prevention. This makes determining the role of climate inherently difficult, but as climate change becomes more pronounced and has a larger impact on the environment, there is little doubt that this will affect the geographical distribution of diseases.

This paper presents a brief literature review of the role of climate, environmental and societal factors in different stages of tick lifecycle and reindeer management that may play a role in the distribution and migration of TBDs in reindeer, in order to establish (1) changes in the occurrence of ticks, hosts and TBDs, and (2) specific links to climate variables. We focus on the Nordics, including Norway, Sweden and Finland, but use especially Norway as an example case. For the literature search, we used the ORIA database available via the University of Oslo Library, which includes all major scientific literature databases, such as Medline, PubMed, BioMed, Web of Science, SCOPUS, ScienceDirect, etc. The following general syntax was employed, in English and Norwegian, including references to TBDs, climate and the focus area: “CSI (e.g., Anaplasm * OR Babesi * OR Tularemi *) AND Climat * AND (North * OR Nord * OR Norw *)”. We also did a similar search, substituting the disease for tick occurrence, with and without references to the target area. The identified literature was scanned for the necessary environmental conditions for the survival and/or activity either of the disease, the vector or the host.

### 2.1. Current Distribution, Lifecycle and Spatio-Temporal Vulnerabilities for Tick-Borne Infections

Because *I. ricinus* spends most of its life cycle in the external environment rather than on its hosts, ticks are highly exposed to the prevailing weather [12]. Thus, direct and indirect (e.g., via vegetation changes) climate change effects on the environment are likely to affect tick survival, development and reproduction, while climate effects will impact both vector (tick) abundance and distribution, and host numbers, migration patterns and diversity. Slow changes in climate, vegetation and host densities are likely to expand both abundance and activity onset in southern regions in the Nordics, and distribution opportunities north for ticks, while an increased number of extreme events and short-term weather variations may limit their abundance and distribution potential.

**Figure 1:**
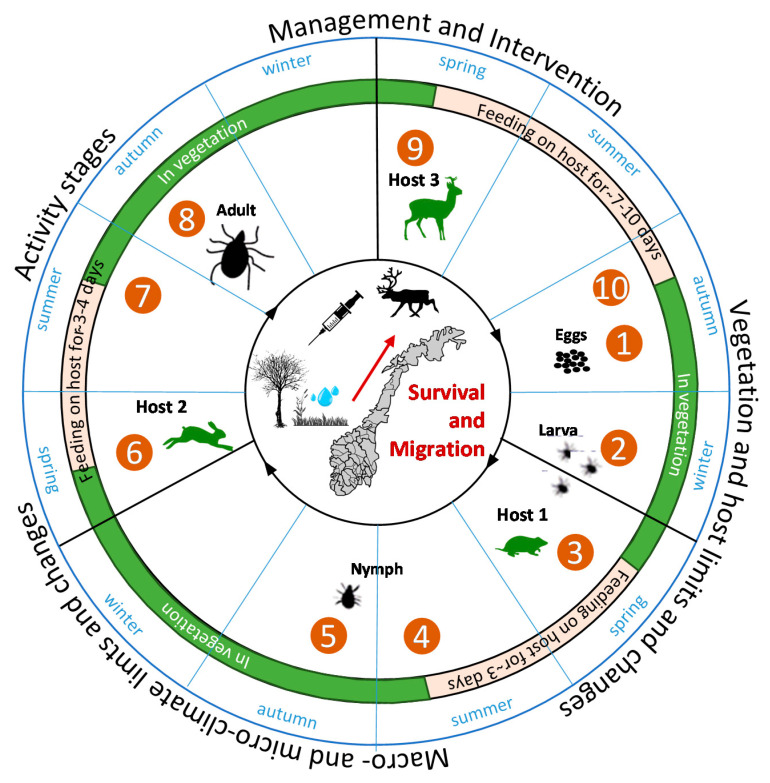
The *Ixodes ricinus* (tick) life cycle and linkages to its environment: Development, inactive (overwintering) and active (questing and on host) stages, climate limits and changes, preferred hosts and vegetation, and management and interventions all form boundaries for the survival and migration of ticks and the CSIs they carry. Descriptive cycle text nos. 1–10 based on www.cdc.gov/dpdx/ticks/index.html.

The *I. ricinus* life cycle usually spans two to three years, but can take up to six years, and includes three hosts (Figure 1), which can be different or the same species. Ticks go through four life stages: egg, larvae, nymph and adult. Adult females drop off the third host to lay eggs after feeding, usually in autumn (1). Eggs hatch into larvae (2), which overwinter in the vegetation. In spring to early summer, the larvae quest and attach to the first host to feed for about 2–3 days, commonly a small mammal or bird (3). By late summer, engorged larvae have dropped off the first host into the vegetation and (4) molt into nymphs, usually in autumn. Nymphs overwinter in the vegetation and the following spring they quest and attach to the second host (6), again usually a small mammal. The nymphs feed on the second host for a period of around 3 to 4 days before dropping back into the vegetation again (7) to molt into adults in late summer/autumn, and overwinter in the vegetation (8). Humans are most commonly bitten by nymphs. Next spring, adult ticks seek out and attach to a third host, usually a large to medium sized mammal (including cervids and bovids) (9). Males feed for a brief period of several hours, while females normally feed only once. The sexes couple whenever they encounter each other, mostly on the hosts, and drop off the host at the latest by autumn (10) to continue the cycle. Females die upon completing oviposition.

The current distribution of ticks and ABT is constrained (or enabled) by many environmental and critical climatic conditions, which may reduce or stop the functioning of the mechanisms involved in the transmission of the disease, as well as its reservoir, reduce activity, or kill the vectors (i.e., ticks) or hosts (e.g., rodents or deer) at various life-cycle stages. Critical limits may also pertain to conditions in the surrounding environment, such as types of vegetation or microclimates (temperature and humidity) where hosts do not thrive, or find food, shade or protection [27]. The combined climatic, environmental and host presence requirements link ticks primarily to the coastal areas in Norway, but also inland, while in Sweden and Finland *I. ricinus* mostly occurs in the south-to-middle of these countries [28]. In Norway, the preferred vegetation is denser and preferably shaded, deciduous or mixed or fragmented forest types in general, while the highest tick densities in Sweden are in the broad-leaf vegetation types and mixed deciduous woodlands [27]. Ticks occur less in pine forest and least in grass and heath, explained by ticks being exposed to more desiccating weather conditions in open vegetation [27,29]. Several studies have linked the presence of ticks with the incidence of pathogens in ticks, humans, and animals [11,30,31,32]. In general, the findings confirm that not all, but varying percentages of ticks are carriers of the pathogens, sometimes more in females and in nymph stages. An increased emergence of Lyme disease, babesiosis and anaplasmosis both in time and space was found, meaning that an increase in both distribution and incidence of diseases had occurred during the period 1995–2015 [31]. Importantly, the prevalence of the pathogens in ticks and the incidence of the diseases in cattle and humans were both spatially and temporally correlated with (changing) exposure with regards to anaplasmosis and babesiosis, while the incidence of anaplasmosis in sheep was only weakly linked with exposure [31].

### 2.2. Ticks and Climate Requirements: Results from Literature Review

Table 1 presents the climatic variables, and identified limits and optimal conditions, for different development stages and activity phases for ticks. There is no consensus on which factors are the most important for tick presence and abundance, but most studies suggest that the tick host-seeking activity (questing), which is particularly important in light of disease transmission risk, has a lower limit of 7–8 °C daily maximum air temperature [33,34,35] for adults, or 10 °C for larvae [12]. While ticks may survive temperatures as low as −18.9 °C or −10 °C for periods of time, these limits do not allow for tick activity, and are thus far below transmission risk. Upper limits are related to both temperature and humidity. Qviller et al. [12] find that questing tick densities decrease above 15–17 °C, while Gray [34] showed that questing persists throughout hot dry weather at temperatures up to 35 °C, as long as appropriate vegetation cover is present to provide opportunities for rehydration. There is also evidence that summer temperatures above 30 °C can change seasonal host-seeking activity patterns and induce diapause, causing a large proportion of the tick population to become more abundant in autumn/early winter than in spring [26,36,37], thus affecting the timing of disease transmission risk. Large day-to-day fluctuations in ground surface temperature in general, and in certain months of the year, and seasonal variation in precipitation changes were found to be associated with the presence/absence of *Ixodes ricinus* and the pathogen *Anaplasma phagocytophilum*, and such an association is found as well in other vectors and diseases such as malaria [20]. Climate change-related changes in the absolute levels of temperature and precipitation are offset by an increase in temperature and precipitation variability, and this might be relevant not only for vector distribution, but also disease transmission [38].

Perhaps the most critical climatic condition for tick survival is a relative humidity of at least 80%, to prevent deadly desiccation of the free-living stages, restricting *I. ricinus* to areas of moderate to high rainfall with good vegetation cover and soil surface humidity through the driest times of the year [27,37,48,49]. While many studies identified temperature links with tick survival and activity, the differences in findings underline that it is not straightforward to extrapolate from seasonal temperature conditions to survival and activity, since ticks experience other temperatures and humidity conditions in the microsites (i.e., leaf litter and soil, under snow [50]).

Jaenson and Lindgren [39] found that in Sweden, tick distribution corresponded most closely to the areas representing an early start of the growing season (before May 1st) and a vegetation period length of about 170 days, and that the length of the vegetation period is determined by similar daily mean temperatures above 5 °C. Jore et al. [20], on the other hand, suggest that the prominent coastal distribution of *I. ricinus* in Norway is better explained by the length of the period without snow than the length of the growth period, as ticks overwinter on the ground and survival can be enhanced by an insulating snow cover layer, which prevents the ground temperature from dropping below zero. Cold winters and a lack of snow may also affect the survival of small mammal hosts the following year, which could mean fewer blood hosts for ticks. *I. ricinus* was consistently present when the period of snow cover was less than or equal to 125 days per year, and was consistently absent with a snow cover greater than or equal to 175 days per year.

## 3. What Does a Changing Climate Mean for Ticks and CSIs?

The climate is changing more rapidly in the Arctic than elsewhere. In the Nordic countries, annual average temperatures are currently 1.5 °C above the normal, and have consistently been warmer since the 1990s. Winter temperatures have increased by about 2 °C, while spring and autumn are about 1.5 °C warmer than normal (www.met.no/vaer-og-klima/klima-siste-150-ar). Precipitation has likewise increased in the last 35 years, especially in spring and winter, and more recently also summer. The snow season is shorter, precipitation increasingly falls in the form of rain, and an increased number of freeze–thaw events means changes in hydrology, type and access to vegetation. Since 1965, the temperature increase in Norway has been 0.3–0.4 °C per decade. This is almost three times faster than the global increase during the same period [51]. In Sweden, temperatures in the last 30–35 years have increased compared to the 1961–1990 normal, especially for winter and spring (by about 1.5 °C and 2 °C, respectively). Coastal Sweden is dryer than inland, on the Norwegian border, but all of Sweden has seen an increase in precipitation over the last 30 years (www.smhi.se/klimat/framtidens-klimat). A study of climate change and extremes in the Nordic countries finds that the observed temperature changes are greatest for daily minimum temperatures. Important for tick activity and development, the area with a growing season over 180 days has increased, from 37,000 km^2^ for the period 1971–2000, to 45,000 km^2^ for the period 1985–2014 [51], and increased especially in coastal areas and the north of Norway, reaching up to 180 days close to 66–67° N.

### 3.1. Changing Distribution Attributed to Climate Change

**Figure 2:**
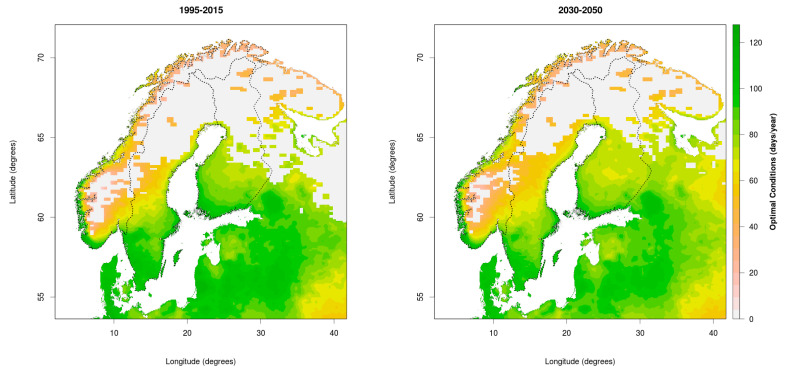
Mean number of days per year from March to November with optimal climatic conditions for tick development for past/present (1995–2015) and future (2030–2050).

**Figure 3:**
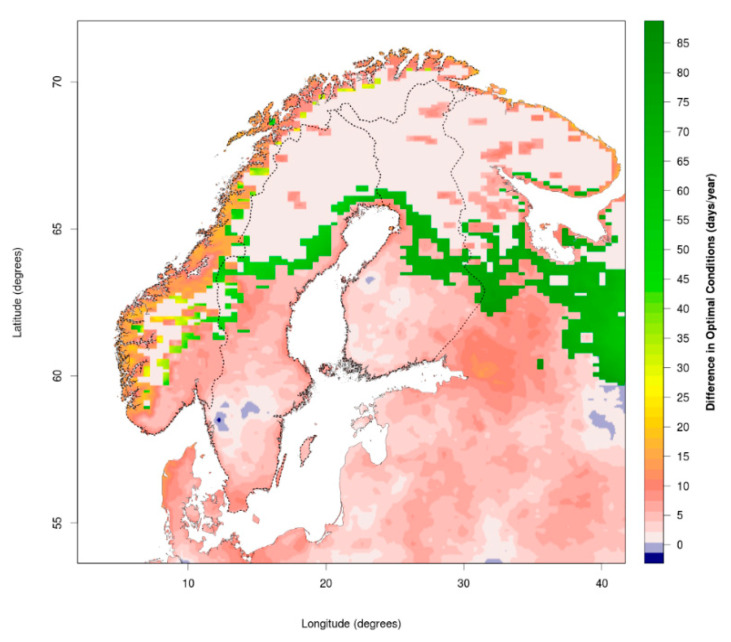
Difference in number of days/year between past/present and future (RCP4.5) optimal climatic conditions.

In our analysis of potential climate change consequences for CSI distribution, we select climatic limits from Table 1 corresponding to tick activity, assuming inactive ticks would not be able to transmit the pathogens. The selected constraints include: (1) daily mean temperature between 7 °C and 35 °C; (2) daily relative humidity equal or above 80% allows tick presence; and (3) more than 175 snow cover days per year (all months) excludes tick presence. Figure 2 and Figure 3 present the number of days in which these conditions are met in both past/present (1995–2015) and future (2030–2050). Regionally downscaled geospatial meteorological data (resolution 0.11° × 0.11°) from the CORDEX project are calculated using the NorESM1-M (CMIP5) output for historical, and RCP4.5 scenarios for future climate conditions. Not included in these figures, but discussed in the paper, are other variables such as vegetation type, presence of host animals, management, etc., which play a further role in the optimal conditions for tick presence.

For 1995–2015, the mean number of days per year with optimal conditions was 29.3, 39.0 and 39.8 for Norway, Sweden and Finland respectively. In future (2030–2050) RCP4.5 scenario climate conditions, the mean numbers of days per year increase to 39.0, 46.4 and 49.7, respectively, accounting for rises of 33.1%, 19.0% and 24.8%. Increased optimal conditions in Norway are mainly at the coast, dominated by a 2–3-week increase in season (Figure 3; most but not all red and orange areas), and less for range expansion (Figure 3; especially the green areas). In Sweden and Finland, the figures show both a range expansion and increase in season. The increased areas with optimal climatic conditions are 9.2%, 9.4% and 11.4% of Norway, Sweden and Finland, respectively. However, some of these new areas may still have too short a season to allow realistic tick settlement.

### 3.2. Comparing Current and Potential Future Distribution to Observations

The warmer and wetter weather trends throughout Fenno-Scandia, especially for spring and winter, facilitate migration towards the north and a longer tick season in the south. The effect of precipitation, however, is probably not a linear one; there seems to be a positive effect at lower and moderate levels, but a negative relationship is seen with higher values (>100 mm per month) [20]. At high latitudes, summer temperatures may still be too low to complete tick development before the onset of winter, but particularly changes in the 7 °C questing limit, along with increased precipitation in winter and spring, improve the climate for tick presence and/or intensity of their activity, and elevate the risks for tick settlement and diseases transmission increasingly further north. An important consideration here is that while annual and seasonal climate trends might indicate increased distribution and abundance potential for ticks and hosts, extreme weather events, especially large fluctuations in temperature combined with hot and dry weather, may represent limits to tick survival when they are not protected by host or vegetation, but the effects seem to be highly dependent on season of occurrence, and in some instances could have a positive effect on tick distribution [20,36]. The effect of the reduced snow cover observed in the last 30 years in Norway, and the projected reduction in snow cover [52], could be a limiting factor for tick and pathogen distribution [20].

Vegetation co-determines both relative humidity and host occurrence, and climatic changes are causing large vegetational shifts, such as shrub encroachment, which is increasingly providing more suitable habitats for ticks and hosts [20,53]. Our projections for suitable habitats derived from climate variables show a major overlap with similar analyses of the potential distribution of ticks based on the growing season and vegetation period [39]. While vegetation is determined by climatic variables, it is also affected by the activity and density of grazers and browsers. Thus, vegetation is only a rough indicator, because the movement of animals between grass and woodlands also affects the distribution of ticks, complicating assessments of preferred vegetation [20]. Compared to the 1980s, some coastal areas in northern Norway are greening as early as April due to an earlier spring [54,55], while shrub encroachment on tundra is increasing. Earlier greening allows geese to arrive earlier to their resting sites along northern coasts, though the role of geese in carrying infected ticks, specifically for ABT, is uncertain [56]. This illustrates that tick range expansion may be facilitated by hosts who are just carriers and not necessarily infected themselves. Moreover, animals such as geese may cross mountains and other geophysical boundaries to reach suitable settlement areas further north. As the climate (Figure 2 and Figure 3) and vegetation [39] change, we get a better idea of the risk picture for the spread of infectious pathogens.

Several Nordic studies on ticks and TBDs have shown that *I. ricinus* has changed its distribution in recent decennia [20,45,57,58], with a northward migration of approximately 400 km within the past 20–30 years in Norway [58,59], up to 150–250 km north of the Arctic Circle, though with a sparser presence north of 65° N [60,61]. A recent study by Hvidsten et al. [45] found that the northernmost permanent *I. ricinus* population was located at 66.22° N at the coast, although this study did not have national coverage and used only one method (flagging) with low efficiency, and cannot therefore be used to verify presence or absence [32]. Nevertheless, the study suggests presence at a high latitude, and the range expansion indicated in the area of 69° N (northern tip of Sweden) is in line with the distributional limit predicted previously [20,28,31]. Tick capture experiments and antibody measurements in sheep blood samples by Jore et al. [20] showed an overall increasing prevalence of *Anaplasma phagocytophilum* in sheep during the last 30 years, both on farmland and rough pasture areas in southern Norway. Antibody presence is not equal to presence of diseases, but may nevertheless suggest changes in tick exposure. This suggests that (more heat and drought sensitive) open vegetation, such as farmland, may become more habitable. Both in Sweden and Norway, increased *I. ricinus* abundance and TBD risk was associated with shrub encroachment and abandonment of fields [20,27], while in Norway the increase of TBDs seems to be greater at the coast, where humidity and spring/autumn temperatures are higher than further inland. The observations of tick distribution match well with the projections shown in Figure 2 and Figure 3, and the increased climatic potential for tick dispersal is further matched by observations of increased TBD incidence in both humans and animals in these regions, documented both in scientific studies [31,62] and national reporting databases on TBDs (e.g., Reporting System for Infectious Diseases MSIS www.fhi.no/hn/helseregistre-og-registre/msis/, or the tick-center flåttsenteret.no). However, as described in a recent study by Jore et al. on human subjects [32], the relationship between tick bites and the risk of tick-borne diseases is poorly understood, since only a fraction of bites lead to disease, and those who develop diseases are not all diagnosed. Thus, the presence of ticks and the risk of TBDs are two different issues. The study by Hvidsten et al. [45] underlined this by investigating the prevalence of *Borrelia* spp. in the northernmost population of *I. ricinus*, having found a low prevalence of about 1–15%, compared to 15–27% in more southern Trøndelag populations. The prevalence of *Rickettsia* spp. in this population was even lower (around 1%). This suggests that while ticks may be expanding their range, the prevalence of TBDs is not equally high in all populations, and may be lower in northern populations.

## 4. Effects of Societal and Structural Factors on Tick Occurrences

While temperature, humidity, snow cover duration, and the changing abundance and availability of host species (especially red deer) and vegetation are amongst the determining factors for tick distribution [20,30], animal and land management variables, such as farm density, shrub encroachment and changing land cover, are also of key importance [20]. Land-use change is considered one of the most important reasons for the emergence of TBDs [20,63], indicating that rationalization processes and structural changes in agriculture, following changed agricultural practices, presently have a strong effect on tick survival and dispersion. In Norway, the number of grazing animals in outfield pastures has been reduced by close to 50% over the past six decades, while wild cervid populations have increased dramatically [64]. This gradual change from grazing livestock to browsing herbivores, attributed to climatic and vegetation changes including shrub encroachment of previous pasture lands and coastal meadows, increases the abundance of ticks [20,29,65].

Many regions in Europe are currently undergoing rapid alterations in land-use, and diminishing livestock-pasture management and fodder harvesting have resulted in ongoing shrub encroachment across vast areas [29,64,65]. Some of the increased forest cover is intentional, and subsidized through EU reforestation schemes to improve ecosystem services, such as biodiversity and carbon sequestration [29]. However, landscape management practices, such as woodland expansion and restoration, urban greening and implementing biodiversity policies, can also have unintended effects on vector-borne pathogen transmission, by increasing the suitable habitat for hosts and the tick vectors [66]. Thus, both the types and levels of human activities, active management and unintended land cover changes, and changes in the presence of domestic species (and their interactions with wild species) can have consequences for TBD incidence [63].

### 4.1. Ticks Are a Management and Policy Issue

As outlined, there are highly complex, cumulative and partially unknown linkages between tick presence (density), the permanence of their presence and the prevalence of TBDs in these populations, related to climate change and land-use practices. While the increasing number of ticks in new areas can be explained by biotic and abiotic factors, and host behavior and abundance [36], the presence and migration of host animals and climate change (including climate variability and the intensity of human exposure (through activities)) also have profound effects on the incidence of tick-borne diseases. Vegetation changes are caused by climate, ecological and human land-use changes, and their interactions. Land-use change is in turn driven by a combination of environmental, political and socio-economic conditions, or reduced access for reindeer to migration routes due to restrictions to border crossings (between Norway and Sweden). Ongoing trends in agricultural and pastoral industries toward larger units with higher production yields have resulted in fundamental changes in animal husbandry both in Norway and internationally [67]. Traditionally, grazing livestock have kept woody shrubs down for centuries, and in Norway, sheep often grazed in the low-lying cultivated infields and mountainous commons, unlike in recent times, where sheep commonly graze at higher altitudes [68]. At present, a high risk of predators reduces the use of certain pastures, which combines with increasing pressure from infrastructural encroachments and human activity exacerbating shrubification [68,69]. These conditions must be seen in connection with the decreasing financial viability of the sheep farming sector, leading to fewer farms and in turn decreased grazing in certain areas. The cumulative and cascading effects of multiple changes illustrates that tick dispersal is a pertinent management and policy issue across agricultural and health sectors.

Gilbert et al. [29] suggest management measures such as grazing to maintain grass cover and thereby reduce TBD risk in pastoral landscapes. In addition to such biological tick control, the management of pastures and habitat modification, such as drainage, use of herbicides, controlled burning and shrub clearing, alongside the removal of leaf, litter and forest canopy, are potential measures for reducing tick density [9]. However, such practices will have to be repeated, are labor intensive, and can only reduce the abundance of ticks for a short period, while the risk of tick introduction from other animals or areas remains [9]. Other measures to reduce and prevent livestock exposure to ticks include host animal treatment with acaricide (chemical pesticide) [29], or the separation of livestock (e.g., with fencing) from tick-infested areas and hosts. It is also important to consider the exposure of livestock, such as sheep on pastures in spring or cattle in summer to autumn, in relation to tick questing activity (especially in spring). Finally, there are indications that selective animal breeding strategies—at least in sheep—may reduce tick infestation and infections [70]. Besides a focus on management measures, continued control of the prevalence of pathogens in ticks, and disease data from public health registries, are both important, and remain the main sources for the study of the risk of tick-borne diseases [32]. Studies on pathogen carriage in ticks thus far suggest lower pathogen load in ticks in northern Norway compared to southern Norway [31]. When using prevalence data from ticks, it is important to take into account the limited spatial and temporal coverage, and the potential difference of prevalence in feeding and questing ticks, as making inferences from limited or incomplete data can give an incomplete picture of risk [20].

### 4.2. Potential Risks for Migrating Reindeer

Reindeer traditionally migrate seasonally between coastal and inland pastures during Norwegian reindeer herding [71,72]. Institutional barriers (i.e., Norwegian–Swedish border) have restricted traditional seasonal migration patterns for over 100 years, and multiple environmental and societal stressors, such as icing-thawing events, increasing snowfall in high altitudes, increased encroachment (e.g., through human activity) and higher carnivore populations, are increasingly altering herding practices and reducing herders’ flexibility to move reindeer between seasonal pastures [71]. In Nordland county, coastal pastures have become more suitable for reindeer during winter because milder winters in the last few decades have led to less frost at the coast [73], while the above-mentioned drivers of change also increasingly force reindeer to graze in more low-lying areas, towards the coast [71].

The increase of ticks at higher latitudes, combined with their density/distribution in milder and wetter coastal regions, increases reindeer exposure to ticks and potentially TBDs. Several studies have shown the prevalence of TBDs in domesticated livestock and wild cervids [8,9,29], and fatal cases of *A. phagocytophilum* have been reported in reindeer in Norway [8], an ailment that was experimentally confirmed [74]. Other studies have also identified TBDs in reindeer in Germany [75] and Mongolia [76]. Furthermore, cases of both *A. phagocytophilum* and Babesia have now been reported in domesticated livestock in north Norway [31,77], identifying the spreading and increased risk of TBDs in northern latitudes. Even if not infected, reindeer can be carriers of ticks enabling tick migration, as there are anecdotal reports of a single reindeer being infested with over 400,000 individuals of the tick species *Dermacentor albipictus*, according to Stuen [78]. *I. ricinus* has been found on moose, deer and roe deer in coastal areas also in northern Norway [59], and a cross-over to reindeer is likely. This potential for reindeer as host would add to the challenges in reindeer herding, but it also increases the risk of tick and TBD dispersion to inland habitats when reindeer migrate.

## 5. Conclusions

This paper contributes to the discussion of the increased incidence of CSIs in the north. We agree with earlier recommendations [20] to “consider climatic variables year-round to disentangle important seasonal variation, climatic threshold changes, and climate variability and to consider the broader environmental change, including abiotic and biotic factors”, and [36] approve of the importance of “the identification of the role of particular hosts species in transmission cycles for risk assessment and risk management”. The review and analysis show that ticks, and consequently TBDs, are sensitive to (changes in) climate which, together with several other factors, co-defines their current and future range. Critical factors include temperature and humidity. Snow cover, growing season, vegetation change (as a result of climate change or land-use changes), host migration, and the suitability of new hosts or vectors (either for ticks alone or for TBDs) further play important roles in the changes of tick abundance and range.

Management emerges as an important regulatory tool for ticks and/or the risk of disease transfer. Developing policies for addressing shrub encroachment, and pasture and animal management, are particularly important. The results underscore the need to take a seasonal view of TBD risks, because the factors affecting such risks have seasonal cycles. This includes the following: (1) grazing and migratory (host) animal presence; (2) tick (vector) activity; (3) climate and vegetation; and (4) land and animal management. These cycles may or may not coincide, but it is highly likely that they have consequences for CSIs distribution. Moreover, having ticks in a location does not necessarily mean having the risk of disease in the same area, since this is dependent on the co-existence of pathogens in available hosts. The complexity of reindeer husbandry, along with herd migration across spatiotemporal scales and boundaries, national and regional husbandry management, shrub encroachment and animal–vegetation interactions, all play a role in establishing reindeer as potential new carriers of ticks, or even vectors for TBDs/CSIs and their distribution.

Given the diversity of hosts, and increasing climatic and vegetation changes providing opportunities for ticks and TBDs to settle further north, a Nordic research and management approach to monitor, identify and manage CSIs is critical [79]. Examples of such research networks are already present, e.g., the Scantick Innovation and CLINF projects. Management plans must be coordinated with other land-use management plans related to climate mitigation (e.g., forest, shrub encroachment) or food production (e.g., outfield grazing and local resource-use) to understand and address changes in CSI risks. The different time scales of change and local variations suggest that management plans with different temporal and spatial dimensions are needed. These include a long-term management focus on climate–vegetation changes, the short-term management of animal–vegetation interactions and disease intervention, and a management plan that incorporates and addresses how the rate and magnitude of change affects traditional and Indigenous livelihoods, such as reindeer husbandry and sheep farming.

## Figures and Tables

**Table 1:** Climatic criteria for the absence, prevalence or spread of the focus zoonoses by their vectors.

CSI	Identified Climatic Limits	Sources
Anaplasmosis (NO: Sjodogg) and Babesiosis (NO: Blodpiss)	Temperature below 10 °C no development or eggs −15 °C, −18.9 °C for 24 h and −10 °C for 30 days lethal 5 °C: limit for activity Tavg <5 °C for >170 days 7 °C host-seeking activity (questing) for nymphs and adults 10 °C host-seeking activity (questing) for larvae 6.7 °C growing season mean Relative humidity 80–85% 24 °C = limit for occurrence and activity. 15–17 °C = reduced activity >30 °C induces diapause 35 °C = questing limit Precipitation >90 mm per month in April–May reduces egg deposition.	[12,20,36,39,40,41,42,43,44]
	Growing season duration: 175–180 days; 160 days; 170 days	[39,45]
	Snow cover duration: Below 125 days > present; Above 175 days > absent; Above 150 days > absent	[13,20,23]
Tularemia (NO: Harepest)	Temperature: number of days below −12 °C Growing season duration: 160–180 days Snow cover duration: absent above 175 days of snow cover	[46,47]
